# Supplementary material for: DCT4—A New Member of the Dicarboxylate Transporter Family in C4 Grasses
Source: Genome Biol Evol. 2021 Feb 2;13(2):evaa251. doi: 10.1093/gbe/evaa251 (PMC7883667; doi:10.1093/gbe/evaa251)
Supplement: evaa251_Supplementary_Data [file evaa251_supplementary_data.zip › SUPPLEMENTAL FIGURES LEGEND.docx]

SUPPLEMENTAL FIGURES LEGEND

**Supplemental Figure 1.** Gel image showing that *DCT1* (92 bp PCR product) and *DCT2* (115 bp PCR product) genes are present in all grass species tested.

**Supplemental Figure 2.** Gel image showing the presence or absence of *DCT4* (132 bp PCR product) genes from species lacking genome assemblies. Negative controls were *Z. mays* and *B.* *distachyon*, and positive controls were *S. bicolor* and *S. italica*.
