# Supplementary material for: DCT4—A New Member of the Dicarboxylate Transporter Family in C4 Grasses
Source: Genome Biol Evol. 2021 Feb 2;13(2):evaa251. doi: 10.1093/gbe/evaa251 (PMC7883667; doi:10.1093/gbe/evaa251)
Supplement: evaa251_Supplementary_Data [file evaa251_supplementary_data.zip › Weissmann.et.al.Supplemental.figures.pdf]

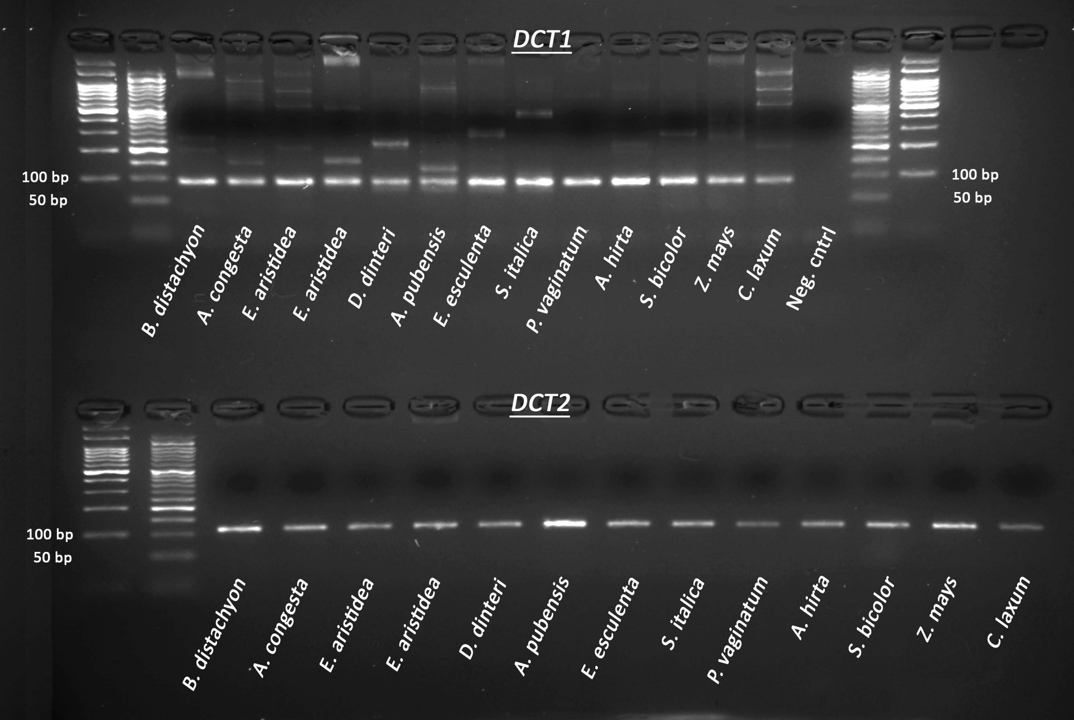
DCT1
100 bp
50 bp
B. distachyon
A. congesta
E. aristidea
E. aristidea
D. dinteri
A. pubensis
E. esculenta
S. italica
P. vaginatum
A. hirta
S. bicolor
Z. mays
C. laxum
Neg. cntrl
100 bp
50 bp
DCT2
100 bp
50 bp
B. distachyon
A. congesta
E. aristidea
E. aristidea
D. dinteri
A. pubensis
E. esculenta
S. italica
P. vaginatum
A. hirta
S. bicolor
Z. mays
C. laxum

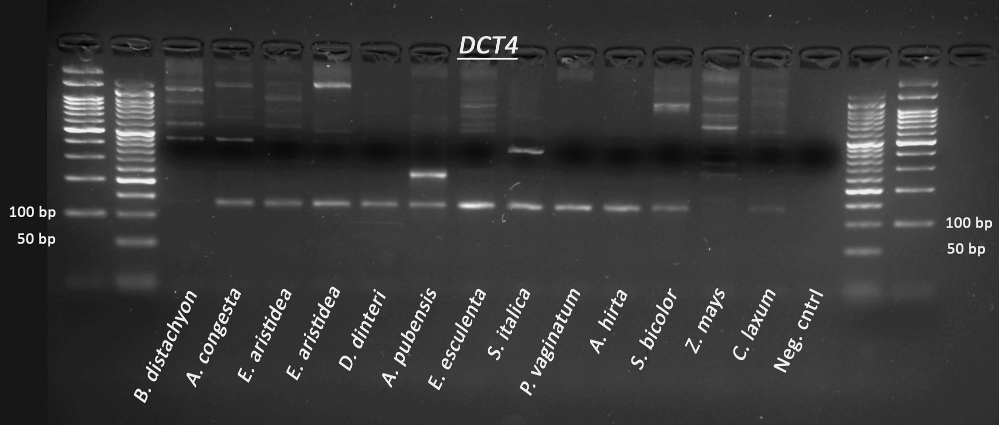
DCT4
100 bp
50 bp
B. distachyon
A. congesta
E. aristidea
E. aristidea
D. dinteri
A. pubensis
E. esculenta
S. italica
P. vaginatum
A. hirta
S. bicolor
Z. mays
C. laxum
Neg. cntrl
100 bp
50 bp
